# Poorly differentiated neuroendocrine rectal carcinoma with uncommon immune-histochemical features and clinical presentation with a subcutaneous metastasis, treated with first line intensive triplet chemotherapy plus bevacizumab FIr-B/FOx regimen: an experience of multidisciplinary management in clinical practice

**DOI:** 10.1186/s12885-019-6214-z

**Published:** 2019-10-16

**Authors:** Gemma Bruera, Antonio Giuliani, Lucia Romano, Alessandro Chiominto, Alessandra Di Sibio, Stefania Mastropietro, Pierluigi Cosenza, Enrico Ricevuto, Mario Schietroma, Francesco Carlei, on behalf of Oncology Network ASL1 Abruzzo

**Affiliations:** 1 Oncology Territorial Care, S. Salvatore Hospital, Oncology Network ASL1 Abruzzo, University of L’Aquila, L’Aquila, Italy; 2 Department of Biotechnological and Applied Clinical Sciences, University of L’Aquila, L’Aquila, Italy; 3 UOC Chirurgia Generale Universitaria, S. Salvatore Hospital, Oncology Network ASL1 Abruzzo, University of L’Aquila, L’Aquila, Italy; 4 UOC Anatomia Patologica, S. Salvatore Hospital, Oncology Network ASL1 Abruzzo, L’Aquila, Italy; 5 Department of Radiology, S. Salvatore Hospital, Oncology Network ASL1 Abruzzo, L’Aquila, Italy; 6 Short Hospitalization Unit, S. Salvatore Hospital, Oncology Network ASL1 Abruzzo, L’Aquila, Italy

**Keywords:** FIr-B/FOx, NEC, Neuroendocrine carcinoma, Thyroid transcription factor-1, Subcutaneous metastasis, Triplet chemotherapy plus bevacizumab

## Abstract

**Background:**

Neuroendocrine tumors (NETs) are heterogeneous, widely distributed tumors arising from neuroendocrine cells. Gastrointestinal (GI)-NETs are the most common and NETs of the rectum represent 15, 2% of gastrointestinal malignancies. Poorly differentiated neuroendocrine carcinomas of the GI tract are uncommon. We report a rare case of poorly differentiated locally advanced rectal neuroendocrine carcinoma with nodal and a subcutaneous metastasis, with a cytoplasmic staining positive for Synaptophysin and Thyroid Transcription Factor-1.

**Case presentation:**

A 72-year-old male presented to hospital, due to lumbar, abdominal, perineal pain, and severe constipation. A whole-body computed tomography scan showed a mass of the right lateral wall of the rectum, determining significant reduction of lumen caliber. It also showed a subcutaneous metastasis of the posterior abdominal wall. Patient underwent a multidisciplinary evaluation, diagnostic and therapeutic plan was shared and defined. The pathological examination of rectal biopsy and subcutaneous nodule revealed features consistent with small-cell poorly differentiated neuroendocrine carcinoma. First line medical treatment with triplet chemotherapy and bevacizumab, according to FIr-B/FOx intensive regimen, administered for the first time in this young elderly patient affected by metastatic rectal NEC was highly active and tolerable, as previously reported in metastatic colo-rectal carcinoma (MCRC). A consistent rapid improvement in clinical conditions were observed during treatment. After 6 cycles of treatment, CT scan and endoscopic evaluation showed clinical complete response of rectal mass and lymph nodes; patient underwent curative surgery confirming the pathologic complete response at PFS 9 months.

**Discussion and conclusions:**

This case report of a locally advanced rectal NEC with an unusual subcutaneous metastasis deserves further investigation of triplet chemotherapy-based intensive regimens in metastatic GEP NEC.

## Background

Neuroendocrine tumors (NETs) are heterogeneous, widely distributed tumors arising from neuroendocrine cells [1–4]. Gastrointestinal (GI)-NETs are the most common (62%) [5, 6], and NETs of the rectum represent 15, 2% of gastrointestinal malignancies [7–9]. Poorly differentiated neuroendocrine carcinomas of the GI tract are uncommon. NETs are defined functioning, if they have the ability to produce peptide hormones, often serotonin, causing the carcinoid syndrome. However, the majority of NETs is non-functioning [10]. The new WHO classification of 2010 [11] distinguishes NETs into well-differentiated and poorly differentiated. Poorly differentiated neuroendocrine carcinomas (NEC) arising from the GI tract are uncommon, but their incidence is increasing. Immunohistochemistry is essential to define the diagnosis, and Chromogranin A (CgA) and Synaptophysin are currently the most specific immunohistochemical markers for NETs [12, 13].

NETs show a metastatic spread in 30% of cases, more commonly liver, while cutaneous metastases are considered rare [14–16]. In clinical practice, relevant bioclinical features addressing the proper multidisciplinary treatment strategy of neuroendocrine carcinoma consist of morphology, Ki-67 expression, mitoses, functional imaging, and clinical behavior.

Even if patients with metastatic high-grade neuroendocrine carcinomas (HGNEC) were prevalently treated with platinum-based chemotherapy, combining cisplatin or carboplatin with etoposide or irinotecan, no standard treatments and clinical management are recommended to date, nor clinical implications according to the primary site of origin, suggesting platinum-based chemotherapy as the treatment of choice [17].

Here we describe a case report of a poorly differentiated NEC originating from rectum, with uncommon immunohistochemical features, and clinical presentation with a subcutaneous metastasis, treated with first line intensive triplet chemotherapy (Oxaliplatin, Irinotecan associated to 5-fluorouracil) plus bevacizumab according to FIr-B/FOx schedule, previously developed by our group, highly active in metastatic colorectal cancer (MCRC) patients, as other reported intensive schedules, such as FOLFOXIRI/bevacizumab [18–20].

## Case presentation

**Fig. 1:**
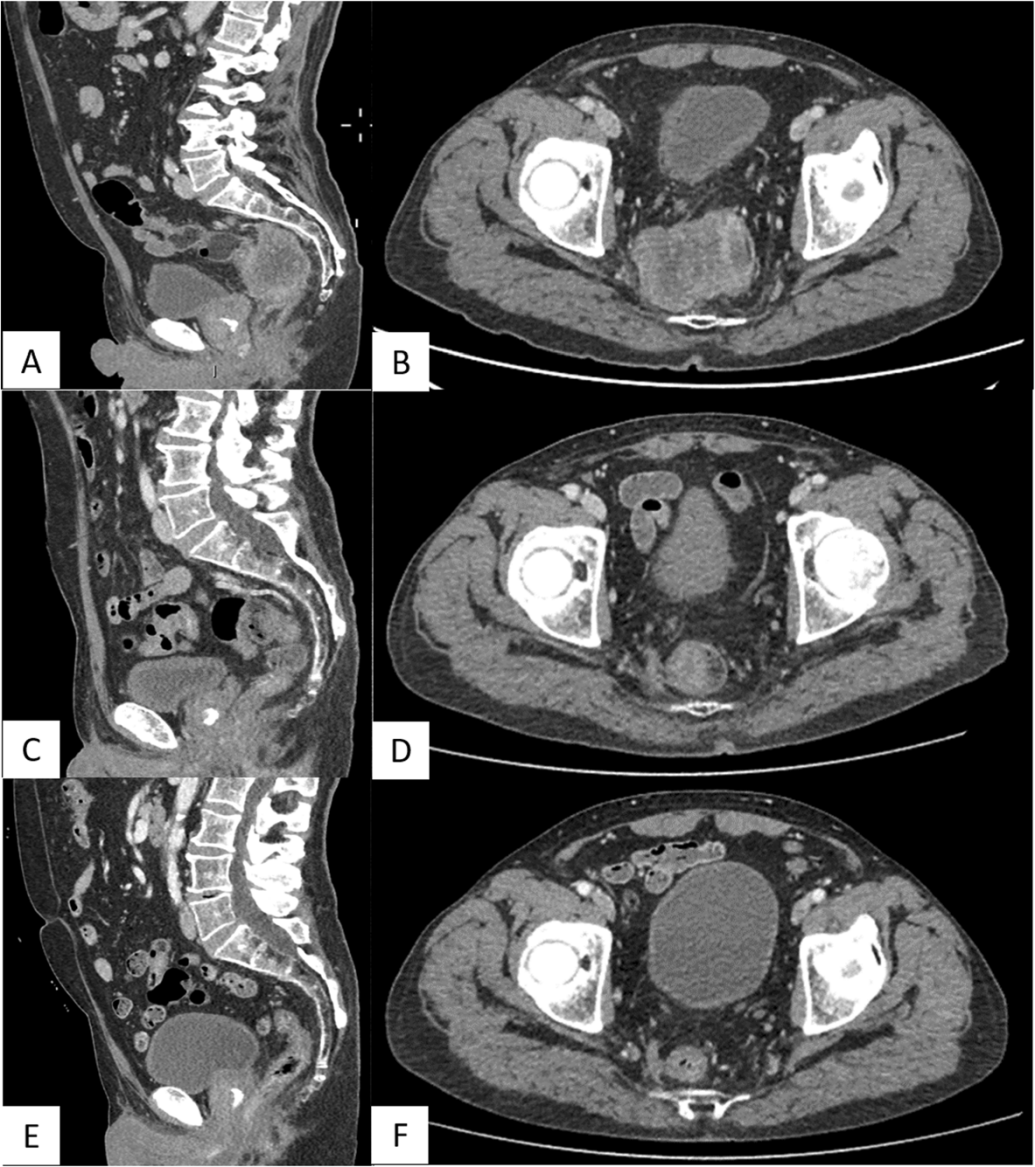
**a**, **b** CT scan showing a mass, centrally colliquated, originating from the right lateral wall of the rectum, infiltrating the right mesorectal fascia, the anterior presacral fascia, the homolateral piriformis muscle, and the right lobe of the prostate gland. The mass caused significant reduction in lumen calibre (**a**, sagittal plain. **b**, axial plain). **c**, **d** Re-evaluation of disease after the first three cycles of treatment. CT scan showed a marked reduction of the rectal mass of about 70–80%, with reduction also of lymph nodes and the prostatic involvement (**c**, sagittal plain. **d**, axial plain). **e**, **f**: CT evaluation after other three cycles of the same medical treatment. It showed further reduction of the rectal mass of about 50%. Lymph nodes and prostatic involvement disappeared (**e**, sagittal plain. **f**, axial plain)

**Fig. 2:**
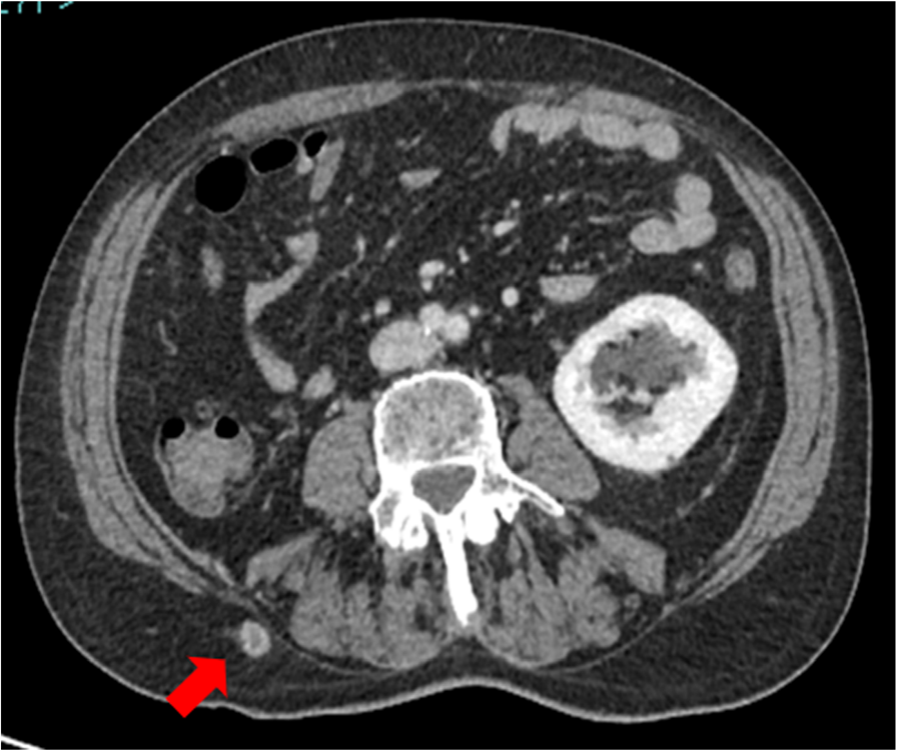
CT scan showed in the context of the subcutaneous soft tissues of the posterior abdominal wall, in the lumbar region, a nodule suspected for a subcutaneous metastasis

A 72-year-old male presented to the Emergency Room of our hospital, due to persistent lumbar, abdominal, perineal pain, and severe constipation. Due to the evidence of increased levels of pancreatic and hepatic enzymes, patient was admitted to a medical ward, and the diagnostic pathway was planned. A whole-body computed tomography (CT) scan was performed (Fig. 1a, b) and showed a mass, centrally colliquated, originating from the right lateral wall of the rectum, with lower margin approximately 7.5 cm far from the anal verge, determining significant reduction in lumen caliber. The mass infiltrated the right mesorectal fascia, posteriorly the right side of the anterior presacral fascia and the postero-medial portions of the homolateral piriformis muscle, and anteriorly the right lobe of the prostate gland. Enlarged lymph nodes suspected for metastatic involvement were detected in the right obturator region (15 mm), along the rectal vessels (10 mm), and in the right side of the prevescical space (10 mm). More, CT scan showed in the context of the subcutaneous soft tissues of the posterior abdominal wall, in the lumbar region, a nodule of 10 mm diameter, centrally colliquated, suspected for a subcutaneous metastasis (Fig. 2).

**Fig. 3:**
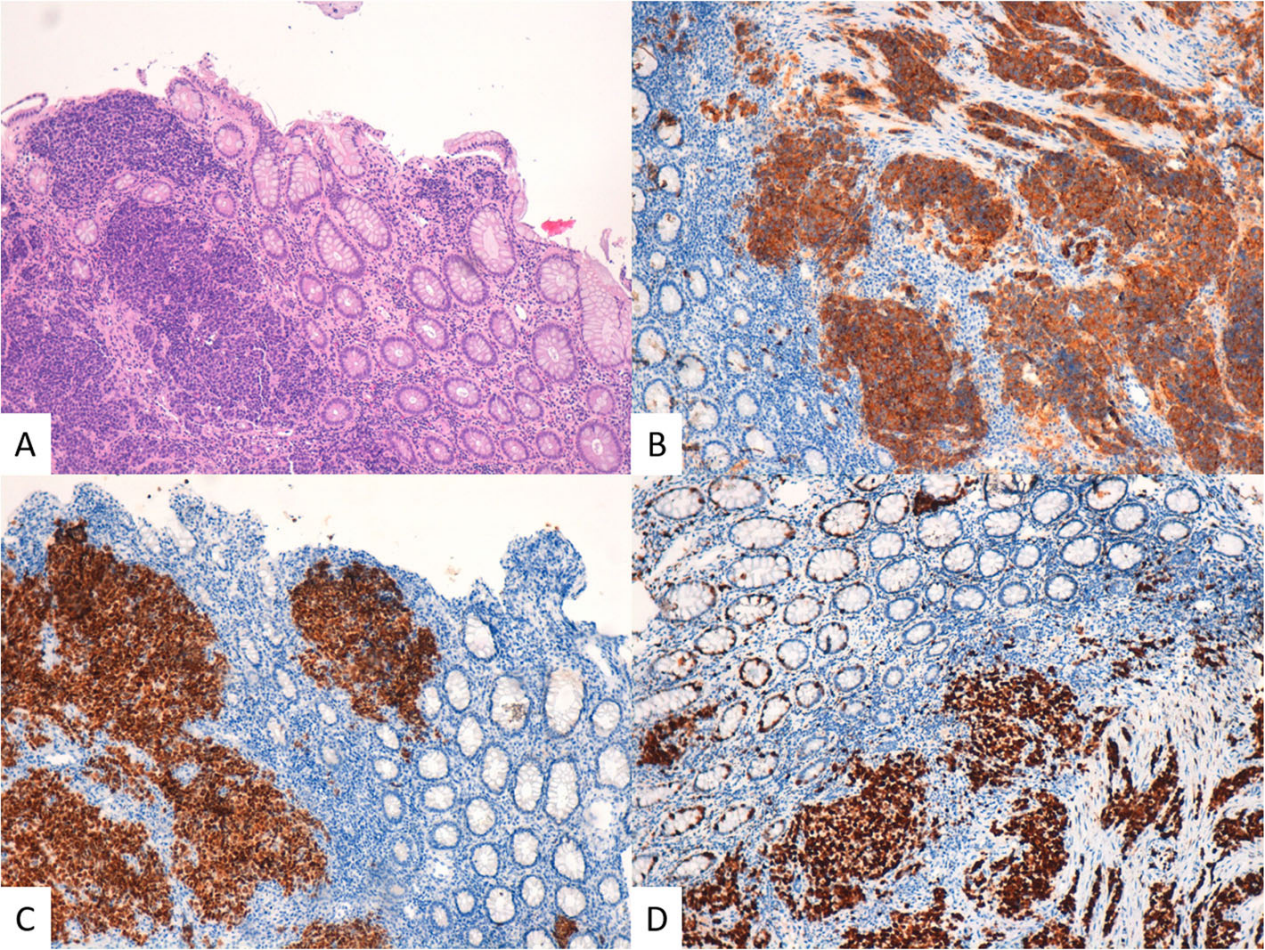
Rectum. **a** The neoplastic tissue infiltrates the mucosa from the bottom up and consists of small cells with scanty cytoplasm (H&E, 100X OM). **b** Synaptophysin IHC (100X OM). **c** TTF1 IHC (100X OM). **d** Ki67 IHC (100X OM)

Patient underwent a multidisciplinary evaluation involving medical oncologist, abdominal surgeon, radiotherapist, and diagnostic and therapeutic plan was shared and defined. Endoscopic evaluation of rectum confirmed an ulcerated proliferation, located 7 cm from the anal verge, involving the bowel semi-circumference, and extending for 5 cm in the cranial direction. The pathological examination of the bioptic specimens revealed features consistent with poorly differentiated NEC, with cytoplasmic staining negative for Chromogranin A and positive for CK AE1/AE3, CD56, Synaptophysin and Thyroid Transcription Factor-1 (TTF-1). Staining for Ki-67 revealed high expression of this proliferation marker in cell nuclei, consistent with a high proliferation rate in tumor cells (Fig. 3). *KRAS*, *NRAS*, and *BRAF* genes were analysed and no mutations were detected.

In the context of soft tissues of the posterior abdominal wall, ultrasound confirmed a hypo-anechoic nodule of 10 mm, suspected for a subcutaneous metastasis.

**Fig. 4:**
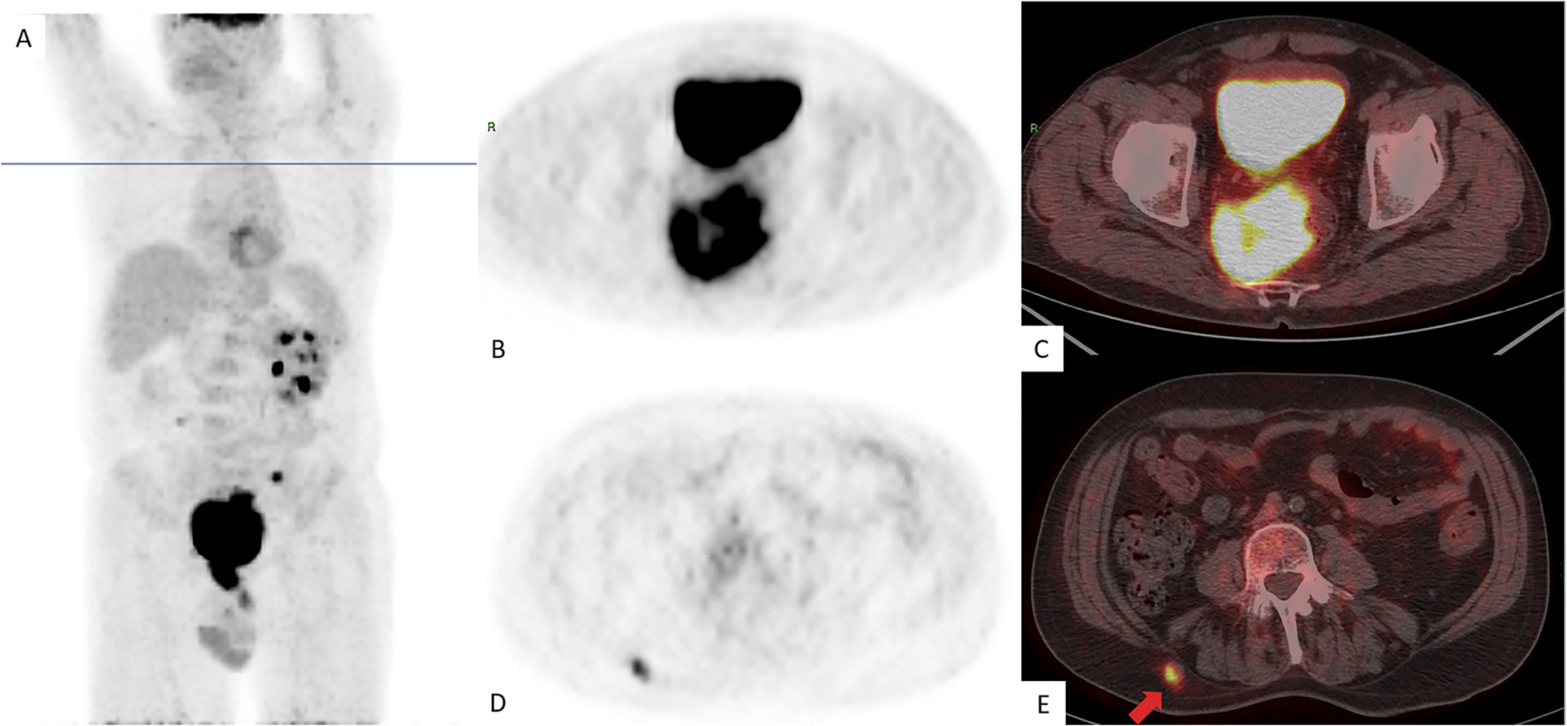
18F-FDG PET showing an extended area of disomogeneous abnormal hypermetabolism at the level of mass of the rectum, with both endoluminal and extraluminal expansion. Coronal plain (**a**), axial plain (**b**) and axial PET/CT (**c**). Pathologic hypermethabolism was confirmed at the level of the subcutaneous nodule of the right lumbar region (**d**, axial PET. **e**, axial PET/CT)

To better define metastatic extension of disease, a whole body 18F-FDG PET was performed (Fig. 4), showing an extended area of disomogeneous abnormal hypermetabolism, probably due to necrotic phenomena, at the level of voluminous mass of the rectum, with both endoluminal and extraluminal expansion, involving the right mesorectal space and reaching the posterior wall of the bladder and the right lobe of the prostate gland, without a well-defined cleavage plan. Pathologic spot was confirmed at the level of lymph nodes and the already known nodule of the subcutaneous soft tissues of the right lumbar region. More, PET showed a metabolic increased concentration at the left sacral wing, near the synchondrosis, with a thickening alteration (Fig. 4).

**Fig. 5:**
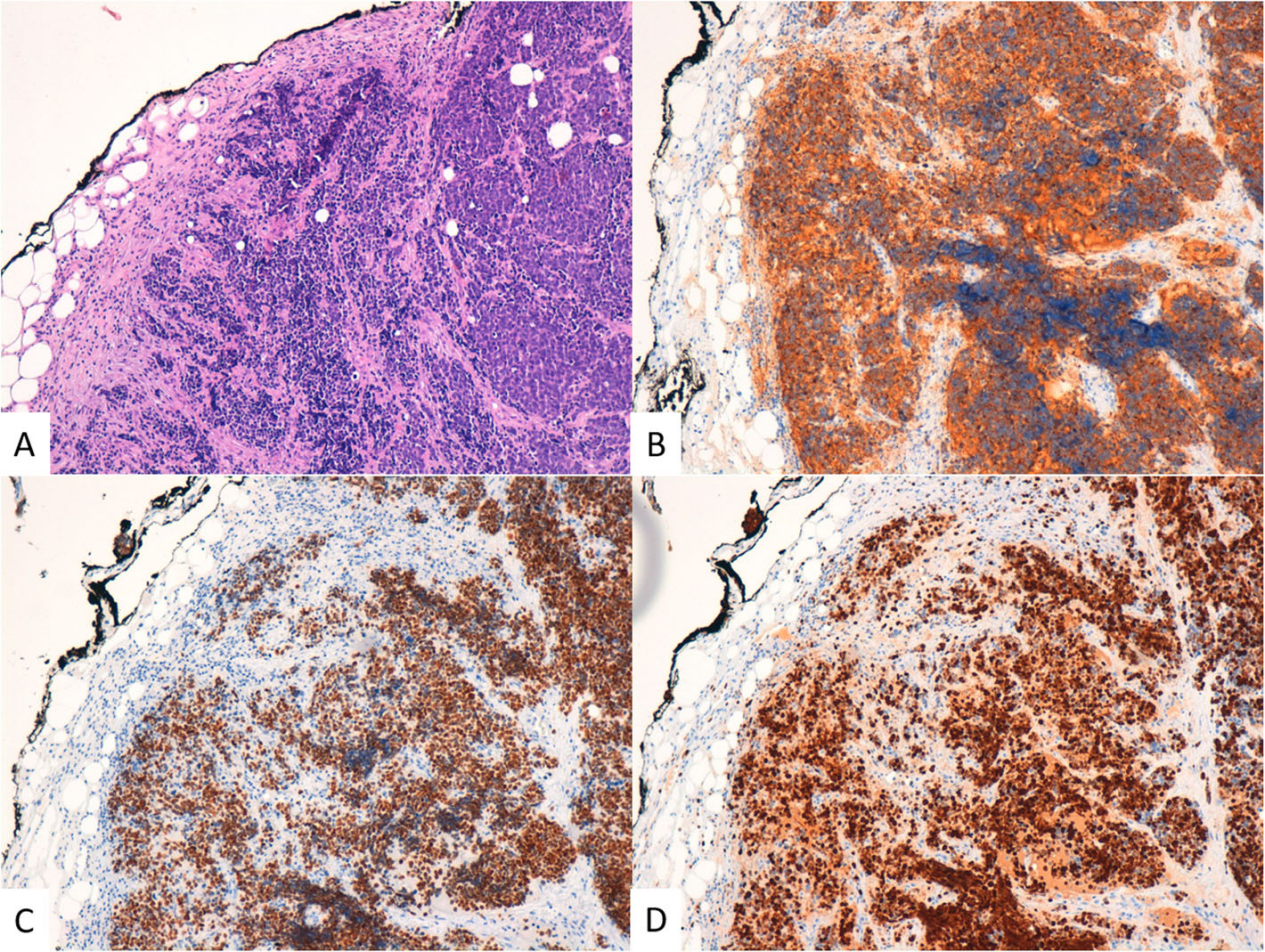
Subcutaneous metastasis. The metastasis shows the same characteristics of the rectal cancer. **a** H&E stain (100X OM). **b** Synaptophysin IHC (100X OM). **c** TTF1 IHC (100X OM). **d** Ki67 IHC (100X OM)

Patient underwent resection of subcutaneous nodule, and the pathological examination revealed features consistent with metastasis from a small-cell NEC. Immunohistochemical study revealed cytoplasmic staining for Synaptophysin, TTF1, AE1/AE3, negative for Chromogranin A, CK20. Staining for proliferation marker Ki-67 was detected in 90% of cell nuclei (Fig. 5). More, patient underwent baseline cardiac evaluation with echocardiogram, showing 70% left ventricular ejection fraction, signs of altered diastolic function, systolic arching of the mitral posterior flap, mild mitral and tricuspid insufficiency, Chiari network in the right atrium.

Laboratory tests, particularly pancreatic and liver enzymes, progressively improved after specifically administered medical treatments; tumor markers, specifically CEA, Ca19.9, Ca125, chromogranin, NSE, and PSA values were in the normal range. Patient underwent an analgesic therapy with pregabalin 75 mg twice a day, and oxycodone naloxone 10 mg twice a day.

Due to metastatic disease, involving a rare site such as subcutaneous tissue and suspected bone metastasis, and locally infiltrating mesorectal, muscular, pre-sacral tissues, prostate gland, with lymph nodes involvement, multidisciplinary team shared the indication to first line medical treatment. Patient was young-elderly (72 years old), with intermediate Cumulative Illness Rating Scale (CIRS) score [19], due to hypertension on treatment, ECOG performance status (PS) 1, symptomatic for pain and constipation, familial history positive for cholangiocarcinoma diagnosed in the father 75 years old, *KRAS*/*NRAS*/*BRAF* wild-type genotype. Intensive first line treatment according to FIr-B/FOx schedule, previously reported as highly active, also specifically in young elderly MCRC patients, was selected due to metastatic, locally advanced rectal NEC: bevacizumab (5 mg/kg) day 1,15 - irinotecan (160 mg/m^2^) day 1,15 – oxaliplatin (80 mg/m^2^) day 8,22 – 5fluorouracil (900 mg/mq/day) day 1–2, 8–9, 15–16, 22–23, cycles repeated every 28 days [17]. A central venous access, port-a-cath, has been placed.

A consistent rapid improvement in clinical conditions, particularly constipation and pain, were observed during treatment, and patient discontinued symptomatic administered therapies. Treatment was well tolerated, with maximum toxicities represented by G2 diarrhea, G2 asthenia, G2 nausea. Re-evaluation of disease was performed, as planned, after the first three cycles of treatment. CT scan showed a marked reduction of the rectal mass of about 70–80%, with reduction also of lymph nodes and the prostatic involvement (Fig. 1c, d). No evidence of the rectal mass was reported at the endoscopic evaluation. Echocardiogram confirmed mild mitralic insufficiency, with 55% left ventricular ejection fraction.

Due to good safety profile of administered treatment, the marked and rapid improvement of symptoms related to primary tumor, and the metastatic extension of disease, multidisciplinary team shared the indication to continue the same medical treatment for other three cycles. Median received dose-intensity was 100% for all associated drugs. Maximum toxicities were represented by G1 nausea, G1 rhinitis, G1 epistaxis, G1 neurotoxicity, G1 asthenia, G3 alopecia, and G3 neutropenia. CT scan showed further improvement of the marked reduction of the rectal mass, and lymph nodes and prostatic involvement disappeared (Fig. 1e, f). No evidence of the rectal mass was reported at the endoscopic evaluation, with the evidence of a scar. Echocardiogram confirmed mild mitralic insufficiency. Multidisciplinary team shared the indication to curative surgery that confirmed a pathologic complete response. At the level of the residual depressed area of distal rectum, histology revealed no evidence of neoplastic tissue and the presence of atrophic mucosa with fibrosis and lymphocyte infiltration; absence of neoplastic cells was confirmed in 32 resected locoregional lymph nodes; actual PFS is 9 months without evidence of residual disease.

## Discussion and conclusions

The 2010 WHO classification defined poorly differentiated neuroendocrine carcinomas (NECs) [8], distinguished into large or small cells [21, 22]. High-grade neuroendocrine carcinomas (HGNECs) have a high mitotic rate (> 20 mitotic figures by 10 high-powered fields, and/or Ki-67 proliferative index > 20%) and show worse prognosis than the more common differentiated neuroendocrine tumors [23–26]. More recently, the definition of G3 NET was introduced to depict pancreatic neuroendocrine neoplasms (NEN) with well differentiated morphology (NET), but with high mitotic rate and/or Ki-67 proliferative index, such as high-grade lesions (WHO 2017).

Incidence of NECs is 1000 cases annually; 11% within the GI tract [27], with a poor prognosis and commonly arising in the oesophagus and large bowel [28–35]. According to the Surveillance, Epidemiology, and End Results (SEER) database, colorectal NECs has an incidence of 0.2 per 100,000 inhabitants [36]. Colorectal small-cell NEC is even more rare [37]. Approximately 200 cases of rectal small-cell carcinoma were reported until present [38].

Pathological diagnosis, including immunohistochemical and molecular markers, is of key relevance: currently, CgA and Synaptophysin are the most specific immunohistochemical markers for NETs. CgA may have limited sensitivity with some of them, such as tumors of the transverse and distal colon, rectum and anus, that have been found to stain in only 20–50% of cases [39–43]. In the present case, tumor had a cytoplasmic staining negative for CgA and positive for Synaptophysin, but it was positive also for TTF-1. TTF1 is a homeodomain-containing nuclear transcription protein of the NK2 homeobox gene family which plays key roles in the control of embryonic development and differentiation [44]. It is involved in the organogenesis of the thyroid gland and lung and in the development of the neurohypophysis and the ventral brain [45, 46]. TTF-1 expression is frequently reported in lung adenocarcinoma (70–80%), while it is negative for virtually all squamous cell carcinomas [47–49]. Lung metastases are usually negative for TTF-1, so it is commonly used to distinguish primary lung adenocarcinomas from tumors of other origin that have metastasized to the lung. TTF-1 could be expressed in typical and atypical carcinoid tumors of the lung [50]. Among neuroendocrine carcinomas of the lung, TTF-1 expression has been reported in 53–100% small cell NECs and 25–75% large cell NECs [50]. Despite the fact that TTF-1 is highly sensitive for small-cell lung carcinomas, its specificity for small-cell carcinomas arising in other sites (i.e. prostate, bladder, uterine cervix, and gastrointestinal tract) is low. Moreover, it is even reported to be uniformly negative in rectal small-cell NEC [51].

In addition to degree of differentiation and proliferation of the tumor, also the presence of metastases or lymph node involvement are reliable markers to predict tumor growth and survival. Approximately 50% of the patients shows synchronous metastatic disease [30, 52, 53]. In an analysis of nationwide Swedish registers by Riihimaki et al. [16] to assess the distribution of metastatic sites of NETs among 7334 patients, 568 with primary rectal cancer, metastatic disease was evident in 1842 patients (25%) and in 71 patients with NETs of the rectum (12.5%). The risk of metastases development was higher in primary tumors of the small intestine or pancreatic-hepatobiliary tract, and lower with appendiceal and rectal NET, and the liver was the most common metastatic site [54–56]. Specifically, among 71 metastatic rectal tumors, 80% involved liver; bone was the second most frequent metastatic site, followed by lung, central nervous system, pleura or mediastinum [16].

Here, we reported a case of a small-cell rectal NEC with a very uncommon, subcutaneous tissue metastatic site. Cutaneous metastases are more commonly present in breast, lung, colon, stomach, uterus and kidney neoplasia [57], while spread to the skin is infrequent in neuroendocrine carcinomas and need to be differentiated from primary neuroendocrine skin tumors, in particular from Merkel cell carcinoma [58–60]. A review conducted by Amorim et al. in 2015 [11] found 31 cases of cutaneous metastases of NET. In most cases, the lesions were painless, single or multiple, non-ulcerated, of slow growth, nodules, and clinically unspecific like other cutaneous metastases. The location of the metastases was most frequently on the cephalic segment and trunk. Only in one case of cutaneous metastasis, primary site was rectum [61], but metastases appeared as multiple subcutaneous nodules, while in our case the metastasis was isolated.

Among gastroenteropancreatic (GEP) NEC, more studies suggested a worse prognosis of colorectal compared to pancreatic NEC [62–64], even if response to chemotherapy seemed to be similar [65, 66].

Ki-67 seemed to play a role to define the proper treatment strategy of GEP and pancreatic NEC. A multicenter, retrospective study of the NORDIC group identified among a population of 305 advanced GEP-NEC (71 pancreatic NEC) treated with first line platinum-based chemotherapy, two subgroups with different prognosis: the former with Ki-67 ≤ 55%, reported objective response rate (ORR) 15%, median overall survival (OS) 14 months; the latter with Ki-67 > 55%, higher ORR 42%, and significantly worse OS 10 months (*p* < 0,001) [65].

A retrospective, multicenter, analysis among 136 patients affected by NEC of different origins, defined three subgroups with different prognosis: well differentiated and Ki-67 20–55%, poorly differentiated and Ki-67% 20–55%, poorly differentiated and Ki-67 > 55%, reporting median OS 43.6, 24.5, and 5.3 months, respectively [67]. Different prognostic relevance was independent from administered treatments. This differentiation suggested that NEC with Ki-67 > 55% may benefit from combination therapies including cisplatin (or carboplatin) and etoposide. Thus, Ki-67%, morphology, functional imaging, clinical behavior, are relevant bioclinical features to define the proper multidisciplinary management and treatment strategy of neuroendocrine carcinoma, in clinical practice.

To date, no standard treatments and clinical management are recommended in high-grade neuroendocrine carcinomas (HGNEC), nor clinical implications according to the primary site of origin, suggesting platinum-based chemotherapy as the treatment of choice [17]. In a retrospective analysis of 100 patients, 89% small cell carcinoma, 60% involving sigmoid or anorectal regions, 64% had metastatic disease at diagnosis, prevalently involving liver. Patients with metastatic disease were prevalently treated with platinum-based associations, combining cisplatin or carboplatin with etoposide or irinotecan, with ORR 42.5%, not significantly different between etoposide and irinotecan based therapies, and median OS 8.7 months. In retrospective analyses, 40% of extrapulmonary HGNEC contained elements of non-neuroendocrine histology, and 30% were associated with an adenoma suggesting the possibility of a common carcinogenic pathway to both adenocarcinomas and HGNEC in colon [17, 68, 69]. Promising clinical outcomes were reported in metastatic poorly differentiated GI-NEC treated with bevacizumab-containing chemotherapy associations, specifically FOLFOX, FOLFIRI and FOLFIRINOX, active in metastatic GI cancers: objective response rate (ORR) 63.6%, DCR 72.7%, median progression-free survival (PFS) 14 months, and median overall survival (OS) 15.3 months [70].

To date, clinical management of metastatic colorectal cancer (MCRC) patients faces with different options and lines of treatment [19, 71], according to patients’ fitness [18, 72–74], extension of metastatic disease [75], and *KRAS*/*NRAS*/*BRAF* genotype [76, 77]. Elderly status (age > 65 years), PS > 2, and/or comorbidities are major features limiting fitness for intensive medical treatments [78].

We previously developed FIr-B/FOx schedule adding bevacizumab (BEV) to triplet chemotherapy, reaching objective response rate (ORR) 82%, progression-free survival (PFS) 12 months, overall survival (OS) 28 months, even effective in young elderly patients [18, 72, 79, 80], not significantly different in *KRAS* exon 2 wild-type and mutant, 38 and 21 months, respectively [19, 76, 77], consistent with those reported by FOLFOXIRI/BEV schedule [81]: median OS 37.1 months in triple wild-type, 25.6 months in *RAS* mutant, 13.4 months in *BRAF* mutant [20]. Among *KRAS*_*2–4*_/*NRAS*_*2–4*_/*BRAF*_*15*_ wild-type and mutant patients treated with FIr-B/FOx, median PFS was 18 and 12 months, median OS 28 and 22 months, respectively, not significantly different [82].

FIr-B/FOx schedule may achieve preferable toxicity profile, particularly in terms of limiting neutropenia, compared to FOLFOXIRI/BEV [18, 72, 73, 78, 79]. In order to more properly evaluate the clinical relevance of toxicity in individual patients, we added the evaluation of individual Limiting Toxicity Syndromes (LTS) [18, 72]. Individual LTS were reported in 46% young-elderly patients, mainly including diarrhea (69.2%), and significantly more represented by LTS-multiple sites compared to LTS-single site, with respect to non-elderly patients [72].

Here, we reported clinical management of a young-elderly patient, with intermediate CIRS stage, PS1, subcutaneous metastasis with lymph nodes and prostatic gland involvement, affected by an undifferentiated rectal carcinoma with neuroendocrine phenotype, *KRAS*/*NRAS*/*BRAF* wild-type, treated with intensive first line FIr-B/FOx regimen. Patient was symptomatic for the presence of primary locally advanced rectal carcinoma, and rapidly experienced a consistent clinical benefit. Received DI was 100% of planned DI, safety profile was acceptable, with no reported LTS. Complete response was reported after 6 cycles of treatment: CT scan showed > 85% reduction of the rectal mass, disappearance of lymph nodes and prostatic involvement; no evidence of the rectal mass at the endoscopic evaluation. Patient underwent curative surgery that confirmed a pathologic complete response without residual neoplastic cells at the level of distal rectum and 32 resected locoregional lymph nodes, at the actual PFS 9 months.

Thus, our present case reported an unusual locally advanced rectal NEC with nodal and a subcutaneous metastasis; FIr-B/FOx intensive regimen administered for the first time in this young- elderly patient affected by metastatic rectal NEC was highly active and tolerable as previously reported in MCRC. This case report confirmed the need for further investigation of triplet chemotherapy-based intensive regimens in metastatic GEP-NEC, reporting high activity and increased clinical outcome in metastatic GI cancers [18, 83, 84].

## Data Availability

The datasets used and/or analysed during the current study are available from the corresponding author on reasonable request.

## Abbreviations

BEV: Bevacizumab
CgA: Chromogranin A
CIRS: Cumulative Illness Rating Scale
CT: Computed tomography
GEP: Gastroenteropancreatic
GI: Gastrointestinal
HGNECs: High-grade neuroendocrine carcinomas
L-L: Liver-limited
LTS: Limiting Toxicity Syndromes
MCRC: Metastatic colorectal cancer
NECs: Neuroendocrine carcinomas
NEN: Neuroendocrine neoplasms
NETs: Neuroendocrine tumors
O/MM: Other/multiple metastatic
ORR: Objective response rate
OS: Overall survival
PFS: Progression-free survival
PS: Performance status
SEER: Surveillance, Epidemiology, and End Results
TTF1: Thyroid Transcription Factor-1
